# Lectures on the Physical Phenomena of Living Beings; Outlines of Human Physiology, for Primary Study and Self-instruction

**Published:** 1848-02

**Authors:** 


*Leçons sur les Phénomènes physiques des corps vivants:* par C. Matteucci. Edition Française publiée avec des Additions considérables sur la deuxième édition Italienne. 18 figures dans le texte. 12mo. pp. 406. Paris: 1847.

*Lectures on the Physical Phenomena of Living Beings.* By Carlo Matteucci, Professor in the University of Pisa. With numerous wood-cuts. Translated under the superintendence of Jonathan Pereira, M. D., F. R. S., Vice President of the Royal Medical and Chirurgical Society. 12mo. pp. 388. Philadelphia: 1848.

*Grundriss der Physiologie des Menschen für das erste Studium und zur Selbstbelehrung.* Von Dr. G. Valentin, ordentlichem Professor der Physiologie und vergleichenden Anatomie an der Universität Bern. Mit zahlreichen in der Text eingedruckten Holzschnitten. 8vo. S. 440. Braunschweig: 1846.

*Outlines of Human Physiology; for primary study and self-instruction.* By Dr. G. Valentin, &c. &c. With numerous wood-cuts in the text. 8vo. pp. 440. Brunswick: 1846.

It is several years since M. Magendie published a work of much interest, under a similar title to that of M. Matteucci. It was translated into English, and appeared in the pages of the London Lancet. It was diffuse; over-speculative, at times; and rambling in its character; but still much interesting information could be gleaned from it; and by us it was regarded as a decided addition to our physiological knowledge—convinced, as we had ever been, that the tendency was—and, with many, still is—to disregard too much the agency of physics in many of the operations of the body; and to refer them all to the unknown quantity *x*—the vital force, under the control of which entity, every functional phenomenon, it was conceived, must be accomplished.

Especially did Magendie dwell on the phenomena of endosmose and exosmose in the healthy, as well as in the diseased condition, and on the importance of our withholding liquids whenever, in morbid cases, the blood escapes from the vessels, either from pathological conditions of their parietes, or by bloodletting under the direction of the practitioner;—inasmuch as the amount of blood circulating in the vessels must be speedily restored if liquids be freely allowed; but it will be blood of a more watery character, and therefore better adapted for transudation through the coats of the vessel; and hence a foundation may be laid for the recurrence of the hemorrhage; when, if the diet had been dry, the results might have been far otherwise. We well recollect the exclamation of Magendie after pointing out the application of these and other facts to the treatment of hæmoptysis. "In presence of such facts, who will still venture to contest the necessity of physical knowledge, if we aspire to practise our profession with superiority!" Yet physical imbibition and transudation are still denied by individuals of position in the medical world!

The name of Matteucci is familiar to many of our readers, in consequence of his experiments and observations on the electrical organs of the torpedo, and on the nervous and electrical phenomena of muscular contraction. The latter are epitomized in the "Physiological Anatomy and Physiology of Man" of Messrs. Todd and Bowman, and their essence has likewise been incorporated into the systems or more complete treatises on physiology. The work before us—both the French and the English version—is a transcript of the Author's lectures, delivered by order of the Government of Tuscany at the University of Pisa. Both profess to have been made under the direction of M. Matteucci. "Dr. Clet"—says the editor of the French edition in his *Avis*—"having executed, under the direction of the author, a translation of the second edition of the *Leçons sur les Phénomènes physiques des Corps vivants*, I hasten to bring this remarkable work to the knowledge of French physiologists." "The present translation"—says the English editor, in an un-English preface—"has been made from a copy furnished to the Editor by Professor Matteucci, and containing a very large number of additions and corrections. Although the French edition is, as far as matter is concerned, more complete than the Italian ones, it contains, on the other hand, numerous errors ascribable to the translator. These have been corrected by M. Matteucci, who has also embodied the results of his most recent investigations in the present edition, which must not therefore be regarded as a mere translation of any of the editions hitherto published."—*Preface*, page v.

There can be little doubt of the greater accuracy of the English copy; but how can we ascribe the errors to the French translator, if the French version was made "under the direction of the author"? For some of the mistakes it is difficult to account; unless the translator is grossly ignorant of the subject. Such, for example, as where we are told at page 126, when treating of "gaseous endosmose" or respiration: that "we must bear in mind the laws discovered by Graham relative to the diffusion of gases in the air; the diffusive powers of gases in air, when they are separated from it by a membrane or by a layer of plaster, *being proportional*" [instead of inversely proportional] "*to the square roots of their density*. The last researches of Valentin and Brunner have verified this law in the phenomena of respiration." This important error is corrected in the English edition, with the following singular explanation, however, in a note by Dr. Pereira:

"In the French edition of Matteucci's lectures, Graham's law is erroneously stated. I have, therefore, corrected the text in the English translation." p. 131.

We do not readily understand the "*therefore*" in this sentence. If the English translation was made from a copy furnished to Dr. Pereira by M. Matteucci, how comes it that so gross an error was not corrected in the copy; and what is meant by correcting "the text" in the English translation? The mistake can hardly be referred "*therefore*" to the French translator.

But the English translation is not without inaccuracies, which the French is free from. In perusing Lectures XIV and XV "on the Nervous Force," we were struck with a physiological authority unknown to us, albeit well versed in such *nomenclature*.

"The physiologists, Bell, Magendie, Müller, *Pandyza*, and others,"—says the English translation—"have discovered that there are some nerves which, when excited, merely produce muscular contractions, and others, which, when submitted to the like irritation, only provoke pain. The anterior and posterior spinal nerves, and some other nervous branches, possess a single function only."

Who was, or is, this *Pandyza?* We suspected this might be a typographical error in the American reprint; but on turning to the English copy, it is there also. In the French version it is correct—*Panizza*—a name known to every physiologist as that of one of the most able and energetic investigators of the phenomena and laws of the animal economy—and more especially of the nerves—of modern times.

We have not done, however, with this sentence. We might properly object to the author for having referred to MM. Müller and Panizza on this subject, unless he had extended the reference to others of his own countrymen, and to numerous observers elsewhere, who have verified and extended the observations of Bell and Magendie, who are pre-eminently entitled to distinction for having first shown, that the anterior roots of the spinal nerves have *motory*,—the posterior roots *sensory* endowments. But we pass to the English translation, and ask for the meaning of the remark, that "the anterior and posterior spinal nerves, and some other nervous branches, *possess a single function only*"? Suspecting some errors in the English version, notwithstanding the assertion of the English editor, that "numerous errors ascribable to the translator" were contained in it, which errors—it will be borne in mind, it is affirmed—"have been corrected by M. Matteucci" in the English version, we turned to the French copy, which is certainly much less obscure.

"The physiologists, Bell, Magendie, Müller, Panizza, and others also, have discovered that there are in the body nerves, which, when excited, provoke only muscular contractility; and others, which, when subjected to the same irritations, provoke pain only. Such is the case with the anterior and posterior roots of the spinal nerves, and with some other nervous branches." p. 259.

Leaving, however, "differential diagnosis" as applicable to the two versions, we proceed to consider the character of M. Matteucci's work, and the matter embraced by it. We have said that it consists of a series of "Lectures." These are twenty in number, and are on the following topics. 1. Introduction. 2. Molecular attraction—Capillarity and Imbibition. 3. Endosmose. 4. Absorption in Animals and Vegetables. 5. Digestion. 6. Respiration—Gaseous Endosmose. 7. Hæmatosis, Nutrition, Animal Heat. 8. Phosphorescence of organized beings. 9. The muscular electric current. 10. Electrical Fishes—Proper current of the frog. 11. Physiological action of Gravity, Light, and Caloric. 12 and 13. Physiological action of the electric current. 14 and 15. Nervous Force. 16. Muscular Contraction—Animal Mechanics. 17. Circulation of the Blood. 18. Vocal apparatus—Voice. 19. Hearing; and 20. Vision. These Lectures are by no means equally well executed. Amongst the best are the third, ninth, twelfth and thirteenth, and fourteenth and fifteenth.

M. Matteucci's conclusions, deduced from his investigations on the subject of endosmose, are as follows:—

"*First.* The membrane interposed between the two liquids is very actively concerned, according to its nature, in the intensity and direction of the endosmotic current.

"*Secondly.* There is, in general, for each membrane a certain position in which endosmose is most intense; and the cases are very rare in which, with fresh membrane, endosmose takes place equally, whatever be the relative position of the membrane to the two liquids.

"*Thirdly.* The direction which is most favourable to endosmose through the skins is usually from the internal to the external surface, with the exception of the skin of the frog, on which endosmose between water and alcohol is promoted from the external to the internal surface.

"*Fourthly.* The direction favourable to endosmose through stomachs and urinary bladders varies much more than with skins, according to the different liquids.

"*Fifthly.* The phenomenon of endosmose is intimately connected with the physiological condition of the membranes.

"*Sixthly.* With membranes dried or altered by putrefaction, either we do not observe the usual difference arising from the position of their surfaces, or endosmose no longer takes place." p. 63.

The following applications of endosmose are full of interest:

"*Endomose applied to Physiology.*—We certainly feel the necessity of having recourse to other experiments in order to exhaust the subject of endosmose, which must play so important a part in all the acts of organized beings. It is certain that the results which we have obtained in this series of experiments, and the view we have taken of endosmose, lead to a more correct application of the phenomenon of endosmose to the functions of organized beings.

"*Endosmose promotes the Mucous Secretion of the Skin.*—The exosmose of a solution of sugar, of albumen, and of gum, towards water, is promoted from the internal to the external surface, in all the skins examined. It is precisely in this same direction through the skin of the torpedo, the eel, the frog, and other animals, that a copious secretion of mucus takes place. The endosmose of water to a solution of sugar, of gum, and of albumen, is less intense from the external to the internal surface of the skin than when it takes place by the reverse arrangement. Consequently, if we do not admit that this secretion of mucus, and this weak absorption of the water wherein these animals live (functions which, for their normal performance, ought always to bear a certain relation to each other,) are entirely due to the phenomena we have discovered; at all events, we cannot deny that they must be promoted by it. Undoubtedly this function of the skin would not go on, or would do so imperfectly, if in these animals which live constantly in water this membrane acted endosmotically in an opposite direction to that which we have found it to do.

"*Endosmose in relation to the Function of the Stomach.*—This constancy observed in the direction most favourable for endosmose and exosmose through skins, does not hold good for the mucous membrane of the stomach of different animals. But every one knows how much more complicated the function of the stomach is, and that all the substances introduced into this organ are either not absorbed, or are absorbed unequally. Moreover, we repeat, that this subject requires elucidation by fresh experiments. When we remark that the direction most favourable to endosmose between water and a saccharine solution, for example, is not the same for the stomach of a ruminant as for the stomach of a carnivorous animal; it clearly follows that the phenomenon of endosmose must be intimately connected with the great differences which exist in the digestive functions of these two orders of animals. I am anxious to explain to you all the details of the experiments which I made with Professor Cima on the subject of endosmose, being well convinced of the great importance of this phenomenon in the vital functions.

"*Ovules nourished and Spermatophora opened by Endosmose.*—It is by endosmose that physiologists now explain the manner in which the nutrition of the ovules in the oviducts of mammalia is effected; and how the sacs, which contain the sperm of the cephalopodus molluscs (or the spermatophora) open immediately they are brought in contact with water.

"*Endosmose of Cells.*—A cell is the elementary organ of all animal and vegetable tissues, and cell-life involves an act of endosmose: this shows how much the phenomenon of endosmose requires to be more completely studied, in order that we may be enabled to make of it all the applications of which it is susceptible.

"*Endosmotic Action of Purgatives.*—I cannot conclude this lecture without referring to the recent experiments of Poiseuille, made with the view of explaining by endosmose the purgative action of certain substances. He found that there was endosmose through animal tissues from the serum of the blood to Seidlitz water, and to solutions of sulphate of soda and common salt. Now this is precisely what happens when we use these medicines internally: the rejected excrements contain an abundant quantity of albumen. In this case we must admit that endosmose takes place through the capillary vessels of the intestine, from the serum of the blood to the saline solution introduced into the alimentary canal.

"*Endosmose of Liquids in Motion.*—But to remove all doubt of the propriety of Poiseuille's applications of this fact, it was necessary to demonstrate that endosmose takes place when one of the liquids is in motion, and is being continually renewed. This has been recently done by Dr. Bachetti, who has shown that the rapidity of endosmose is considerably augmented when one of the liquids was continually renewed. This result, moreover, is in accordance with the principles of the theory of endosmose: the interchange of liquids, constantly going on through the membrane, leads to the suspension of the action of endosmose; or, in other words, the conditions for the production of the phenomenon are so much the better preserved, as the liquids remain longer without mixing. Poiseuille has also shown that endosmose ceases to take place in a membrane after a certain time of action, but that the membrane re-acquires this property by submitting it to the action of other liquids.

"*Remarkable Influence of Morphia.*—The most remarkable fact discovered by Poiseuille is, that of the influence exercised by hydrochlorate of morphia. This substance, when added to saline solution, very considerably weakens the endosmose from the serum to the solution: and ultimately changes the direction of the current. This fact has been confirmed by Dr. Bachetti. How can we make an entire abstraction of this fact in the explanation of the action of morphia, and of the preparations of opium in diarrhœa, as well as of the constipation which they produce?"

The following summary of M. Matteucci's hypothetical views respecting the nervous force, is appended by Dr. Pereira to Lectures XIV and XV.

"1. The nervous fluid is produced by the chemical actions of nutrition.

2. This fluid, developed principally in the muscles, is diffused there, and being endowed with a repulsive force between its parts, like the electric fluid, retains the elements of a muscular fibre in a state of repulsion analogous to that presented by electrified bodies.

3. When this nervous fluid ceases to be free in the muscle, the elements of the muscular fibre mutually attract each other, as we see happens in cadaveric rigidity.

4. This nervous fluid enters continually into the nerves, and from them passes to the brain, assuming in these bodies a new state which is no longer that of the free fluid: in this manner it passes from the muscle to the nerve. According to the quantity of this fluid which ceases to be free in the muscle, the contraction is more or less strong.

5. This state is that of the nervous current, or species of discharge which proceeds from the nervous extremities to the brain, and returns in the contrary direction, by the act of the will.

6. When this discharge takes place, muscular contraction ought to take place, the fluid ceasing to be free in the muscles.

7. This discharge of nervous fluid, acting as in the electric fish, explains the induced contraction; in both cases, and by the same disposition of parts, the nervous current produces a species of electric polarization of the elements, either of the muscular, or of the electric apparatus: the difference of effects will be due to a different number of elements, to their dimensions, &c.

8. The electric current impedes the nervous discharge, if it be directed in the contrary direction; as in the direct current: the nervous fluid not being able to enter and accumulate in the nerve, this loses its excitability. The contrary takes place for the inverse current, which goes in the same direction as that of the nervous discharge: the nervous fluid is found accumulated in the nerve, and its excitability is augmented.—J. P." p. 295.

But we have no space for farther comment. Every portion of the work is well worthy of being perused and studied; and we strongly recommend it to the attention of our readers.

The "*Grundriss der Physiologie des Menschen*" is by the same distinguished physiologist—M. Valentin—as the more elaborate work, entitled "*Lehrbuch der Physiologie des Menschen,*" which was published in Germany, in 1844, in two large volumes octavo. *It*, also, is stated to be intended for physicians and students. The work before us is recommended as a text-book for the beginner in the study of physiology in universities and medico-chirurgical institutions; as a book of reference for physicians, who desire to have, in a small compass, a view of the existing condition of biology, and as a manual for the cultivated laity (*gebildeten Layen*) who may be desirous of becoming acquainted with the human organism. Of the qualifications of the author, who is also one of the most active and accomplished contributors to the "*Handwörterbuch der Physiologie*" of Wagner, there can be no doubt. The work is ably composed, is on good paper, and illustrated by numerous well executed wood-cuts. It is of the same class as, but of a much higher order than, most of the works on popular physiology which we have been accustomed to notice.

